# Revisiting reliability and measurement precision: Towards a unified perspective

**DOI:** 10.1111/bmsp.70068

**Published:** 2026-07-15

**Authors:** Jimmy de la Torre, Klaas Sijtsma, Rodrigo Schames Kreitchmann

**Affiliations:** ^1^ Faculty of Education The University of Hong Kong Hong Kong China; ^2^ Department of Methodology and Statistics TSB Tilburg University Tilburg The Netherlands; ^3^ Department of Methodology of Behavioral Sciences, Faculty of Psychology National University of Distance Education Madrid Spain

**Keywords:** classical test models, cognitive diagnosis models, item response theory, reliability

## Abstract

This article revisits the concepts of reliability and measurement precision across classical test theory (CTT), item response theory (IRT) and cognitive diagnosis models (CDMs), emphasizing their conceptual differences and proposing a unified framework. We show how continuous score estimators in CTT and IRT relate to mastery probabilities in CDMs through integration over proficiency estimator distributions relative to defined thresholds. Building on this link, we introduce reliability indices grounded in the coefficient of determination ($R^{2}$) as a common measure of association between true scores and estimated true scores, applicable to both continuous proficiencies and discrete classifications. This unified perspective clarifies how reliability can be consistently estimated and reported across frameworks, promoting coherence in psychometric practice and supporting more transparent interpretation of test results. Ultimately, this study aims to clarify reliability concepts, facilitating a unified perspective and aiming to promote consistent reliability reporting across diverse psychometric applications.

In the context of classical test theory (CTT), reliability is defined as the proportion of true‐score variation within the variation of observed test scores on the test. This definition is mathematically identical to the squared correlation between the observable score and the true score (Lord & Novick, 1968). Item response theory (IRT) and its multidimensional extension (M‐IRT) model item‐level probabilities as functions of latent variables. Maximum likelihood estimation of the latent variables naturally yields standard errors of estimation and reliability of estimated latent variables as a byproduct. In the context of educational measurement, cognitive diagnosis models (CDMs) assume discrete skill mastery profiles rather than continuous proficiencies represented by latent variables, focusing on the accuracy of categorical classifications. Unlike IRT and M‐IRT, which provide norm‐referenced interpretations, CDMs aim to classify individuals based on their mastery, or lack thereof, of attributes explicitly defined when estimating the models, making them particularly useful for diagnostic assessment.

Although variables of interest in CTT (true score) and IRT (latent variable) are continuous while those in CDMs are discrete (attributes), the mastery probabilities in CDMs can be linked to continuous score estimator distributions. Let $T_{i}$ denote a generic true proficiency score, applicable both in CTT and IRT. Let $x_{i}$ represent the observed data, that is, sum scores in CTT or item scores in IRT. The probability that an individual has truly mastered an attribute can be expressed as the probability that their true score exceeds a mastery threshold τ, $P\left(T>\tau |x_{i}\right)$, approximated by

(1) $$P\left(\alpha =1|x_{i}\right)=\int_{\tau }^{\infty }P\left(T|x_{i}\right)dT,$$

where α=1 denotes a true mastery classification, that is, T>τ (Chen et al., 2018). Classification with respect to multiple attributes requires evaluating multidimensional integrals. For example, for the two‐dimensional profile, $\alpha =\left\{0,1\right\}$, representing non‐mastery of the first attribute and mastery of the second, we have:

(2) $$\begin{array}{c} P\left(\alpha =\left\{0,1\right\}|x_{i}\right)=\int_{-\infty }^{\tau_{1}}\int_{\tau_{2}}^{\infty }P\left(T_{1},T_{2}|x_{i}\right)dT_{1}dT_{2}, \end{array}$$

where $\tau_{1}$ and $\tau_{2}$ denote the mastery thresholds for each attribute.

Equation (2) relates CDM classification probabilities conceptually to the confidence distributions of true scores in CTT and IRT. In this article, we first define the distributions of proficiency estimators within each framework. We then show how mastery probabilities in CDMs and attribute profile probabilities can be related to analogous quantities in CTT and IRT. Finally, we derive an $R^{2}$‐type reliability coefficient for classifications, highlighting its desirable statistical properties and its potential to unify reliability reporting across frameworks. Our unique contribution is to present a unified reliability framework for CTT, IRT, and CDMs. Our approach recognizes the existence of CTT, IRT and CDMs as independent but related approaches to measurement and is different from a more integrated approach discussed by Liu et al. (2025; also, see Sung & Liu, 2026).

## 1 CLASSICAL TEST THEORY

CTT commits to estimating the degree to which random measurement error affects measurement values of any dimensionality (Sijtsma et al., 2024), known as test‐score reliability. Other models, such as factor models and IRT models, typically restrict the dimensionality of the test score, often to one dimension assumed to reflect the attribute of interest. The uniqueness of CTT focusing on reliability of test scores of unknown dimensionality is regularly misunderstood, because we are typically used to restricting the dimensionality of measurement. Moreover, CTT methods to determine reliability of test scores typically are lower bounds to the reliability when the item‐score data are multidimensional, and coincide with reliability when unidimensionality defined as a specific kind of one‐factor model is assumed (Raykov et al., 2023; Sijtsma & Pfadt, 2021). Despite its age and abundant presence in the psychometric literature, different formulations of CTT exist. For example, Liu et al. (2025) and Sung and Liu (2026) discuss a regression approach using a one or more latent variables. Several authors have expressed CTT as a factor model, usually with a dominant factor and group factors (e.g., Bollen, 1989).

We discuss the traditional CTT model without dimensionality assumptions and therefore without latent variables (also see Sijtsma et al., 2024; Sijtsma & Pfadt, 2021; however, see Liu et al., 2025; Sung & Liu, 2026). We focus on the sum score, defined as $X_{+i}=\sum_{j=1}^{J}X_{\mathrm{ji}}$, $X_{j}$ is an item score. CTT (Lord & Novick, 1968) decomposes the sum score (and any other observed score) for person *i* as the sum of a true score ($T_{+i}$) and a measurement error ($e_{+i}$):

(3) $$\begin{array}{c} X_{+i}=T_{+i}+e_{+i}. \end{array}$$

We define the true score on the J‐item test as $T_{+i}=E\left(X_{+i}\right)$, expectation across independent replications of the test administered to person i, but other conceptions are possible (Lord & Novick, 1968, chap. 2). Liu et al. (2025) define the true score as $\tau_{+i}=E\left(X_{+i}|\eta \right)=\sum_{j=1}^{J}P\left(X_{j}=1|\eta \right)$, which ties the true score of person *i* to an underlying latent variable (or variables) *η*, potentially arising from IRT or CDMs, thereby providing an approach that integrates across different measurement models.

The traditional CTT approach implies that across replications, $E\left(e_{+i}\right)=0$, and next, across persons, we have that $E\left(E\left(e_{+i}\right)\right)=0$. CTT assumes that all deviations from this mean are the result of random processes, from which it follows that measurement errors covary zero with true scores in the same and other random variables and measurement errors in other random variables. Then, the variance of observed scores can be decomposed as follows:

(4) $$\begin{array}{c} \sigma_{X_{+}}^{2}=\sigma_{T_{+}}^{2}+\sigma_{e_{+}}^{2}. \end{array}$$

The reliability of the sum score is defined as the proportion of sum‐score variance in the population that is true‐score variance, which can also be interpreted as the proportion of sum‐score variance that is not error variance; that is,

(5) $$\begin{array}{c} \rho_{X_{+}}=\frac{\sigma_{T_{+}}^{2}}{\sigma_{X_{+}}^{2}}=1-\frac{\sigma_{e_{+}}^{2}}{\sigma_{X_{+}}^{2}}. \end{array}$$

Because $\sigma_{T_{+}}^{2}$ and $\sigma_{e_{+}}^{2}$ cannot be estimated directly, CTT has derived several methods that approximate reliability from below, so‐called lower bounds (e.g., Guttman, 1945; Jackson & Agunwamba, 1977; Woodward & Bentler, 1978), coefficient alpha being the best‐known of them and also the most popular in practice.

Henceforth, if no confusion occurs, we write X instead of $X_{+}$ and likewise use simplified notation for other variables based on multiple item scores. Assuming uncorrelated errors and normally distributed measurement error, the distribution of observed scores for a given person i across repeated assessments is normal. The expected value of this *sampling* distribution by definition is $T_{i}$, and its standard deviation is the standard error of measurement for that person, SE(*X*_*i*_|*T*_*i*_). Because the person's propensity distribution (Lord & Novick, 1968, chap. 2) is unknown, in practical applications, the standard error of measurement for person *i* is replaced with its expectation across persons, given by the standard error of measurement (SEM) that is readily derived from the right‐hand side of the definition of reliability:

(6) $$\begin{array}{c} \mathrm{SEM}=\sigma_{e}=\sigma_{X}\sqrt{1-\rho_{X}}. \end{array}$$

Under previously stated standard assumptions of the CTT, the distribution of observed scores for a given person can be expressed as $P\left(X_{i}|T_{i}\right)\sim N\left(E\left(X_{i}\right),\sigma_{e}^{2}\right)$.

Conversely, if parameters $\rho_{X}$ and $\mu_{X}$ are known or precisely estimated, the true score estimator for person *i*, ${\ddot{T}}_{i}$, can be computed based on the linear regression of *T* over *X* (Lord & Novick, 1968, chap. 3), also known as Kelly's estimator:

(7) $$\begin{array}{c} {\ddot{T}}_{i}\left(X_{i}\right)=\rho_{X}X_{i}+\left(1-\rho_{X}\right)\mu_{X}. \end{array}$$

The error of estimation associated with this regression‐based estimate is

(8) $$\begin{array}{c} \epsilon_{i}=\rho_{X}\left(X_{i}-\mu_{X}\right)-\left(T_{i}-\mu_{X}\right), \end{array}$$

and the corresponding standard error of estimation is

(9) $$\begin{array}{c} \sigma_{\epsilon }=\sigma_{X}\sqrt{\rho_{X}}\sqrt{1-\rho_{X}}. \end{array}$$

Because $\sigma_{\epsilon }\leq \sigma_{e}$ when $\rho_{X}$ and $\mu_{X}$ are known or accurately estimated, regression‐based estimates ${\ddot{T}}_{i}$ are more efficient than using observed scores directly but extreme estimates can be biased (van der Ark et al., 2026, studied bias and standard error).

Ignoring consequences of the work of van der Ark et al. (2026), for simplicity, we assume unbiased estimation and estimation errors that are uncorrelated with other variables and express the conditional distribution of a person's true score given an observed score $X_{i}$ as $P\left(T_{i}|X_{i}\right)\sim N\left({\ddot{T}}_{i}\left(X_{i}\right),\sigma_{\epsilon }^{2}\right)$. Accordingly, the probability that a person's true score exceeds a mastery threshold τ is given by

(10) $$\begin{array}{c} P\left(T_{i}>\tau |X_{i}\right)=\int_{\tau }^{\infty }P\left(T_{i}|X_{i}\right)dT=\Phi \left(\frac{{\ddot{T}}_{i}-\tau }{\sigma_{\epsilon }}\;\right), \end{array}$$

where Φ denotes the cumulative standard normal distribution function and τ is a fixed true‐score value (e.g., the minimum true score required for mastery).

When classification involves multiple scale scores, Equation (10) can be generalized as in Equation (2). In that case, the conditional distribution $P\left(T_{i}|X_{i}\right)$ follows a multivariate normal with mean vector ${\ddot{T}}_{i}$ (the regression‐based estimators of person *i*'s true scores across *K* scales) and a diagonal covariance matrix with error variances $\sigma_{\epsilon }$ as entries. For *K* attributes, the classification probability for a mastery profile α is given by

(11) $$\begin{array}{c} P\left(\alpha |X_{i}\right)=\int_{R\left(\alpha \right)}P\left(T_{i}|X_{i}\right)dT, \end{array}$$

where the integration region $R\left(\alpha \right)$ is a hyperrectangle in ${\mathbb{R}}^{K}$, defined by whether each coordinate lies below or above its corresponding mastery threshold. CTT has also defined standard errors conditional on scale values (e.g., Feldt et al., 1985), but we ignore this possibility here.

## 2 ITEM RESPONSE THEORY

In IRT, the person score or parameter, denoted by θ, is a latent variable, in the context of educational testing often a proficiency. Estimation methods for θ are based on the likelihood of item‐score vectors for different levels of *θ*, based on pre‐estimated item parameters collected in the vector **δ**. The three main scoring methods—*maximum likelihood estimation* (MLE), *maximum a posteriori* (MAP) and *expected a posteriori* (EAP)—each provide latent proficiency estimates along with corresponding standard errors (SEs) and reliability measures (e.g. Brown, 2018; Holland & Hoskens, 2003; Kim, 2012). Assuming local independence (i.e. item scores are conditionally independent given *θ*), the likelihood of an observed item‐score pattern for person *i*, denoted by $x_{i}$, is obtained by multiplying the probability of each observed item score given a specific latent variable level $\theta_{q}$ and item parameters **δ**, so that

(12) $$\begin{array}{c} L\left(x_{i}|\theta_{q},\delta \right)=\prod_{j=1}^{J}P\left(x_{\mathrm{ij}}|\theta_{q},\delta \right). \end{array}$$

For person *i*, the MLE method estimate is the value of $\theta_{q}$ that yields the highest likelihood for the observed item response vector $x_{i}$.

The standard error of MLE estimates can be approximated using the inverse of the Fisher information, as follows:

(13) $$\begin{array}{c} \mathrm{SE}\left({\hat{\theta }}_{i}|\theta_{i}\right)=\sqrt{I^{-1}\left(\theta_{i}\right)}, \end{array}$$

where $I\left(\theta_{i}\right)$ represents the test‐level Fisher information about $\theta_{i}$, computed as the sum of item‐level Fisher information values.

The MLE is a consistent estimator of $\theta_{i}$, and its sampling distribution approximates $N\left(\theta_{i},\mathrm{SE}\left({\hat{\theta }}_{i}|\theta_{i}\right)\right)$ as the number of items increases. For short tests or extreme item‐score patterns, the MLE may be biased or even undefined. Throughout this article, let ${\hat{\theta }}_{i}$, ${\overset{˘}{\theta }}_{i}$ and ${\tilde{\theta }}_{i}$ denote MLE, MAP and EAP proficiency estimators. Accordingly, $\mathrm{SE}\left({\hat{\theta }}_{i}|\theta_{i}\right)$, $\mathrm{SE}\left({\overset{˘}{\theta }}_{i}|\theta_{i}\right)$ and $\mathrm{SE}\left({\tilde{\theta }}_{i}|\theta_{i}\right)$ denote the standard errors of the MLE, MAP and EAP estimators, respectively, each calculated using its own appropriate formula. Notably, while MLE and MAP standard errors are derived from the curvature of the likelihood or posterior at the mode, the EAP standard error is based on the posterior standard deviation of the estimators for a specific item‐score pattern. In IRT, $\mathrm{SE}\left({\hat{\theta }}_{i}|\theta_{i}\right)$ varies depending on the interaction between the person's latent proficiency level and the item parameters. In M‐IRT, $I\left(\theta_{i}\right)$ denotes the multivariate information matrix regarding vector $\theta_{i}$ and the sampling distribution of an individual's proficiency estimates follows a multivariate normal distribution, with vector of means of ${\hat{\theta }}_{i}$ and ${\mathrm{Cov}}_{\mathrm{MLE}}\left({\hat{\theta }}_{i}|\theta_{i}\right)=I^{-1}\left(\theta_{i}\right)$.

The estimation error variance across the population, $\sigma_{Ε}^{2}$, is the marginal error variance resulting from integrating the conditional error variances over the ability distribution $g\left(\theta \right)$:

(14) $$\begin{array}{c} \sigma_{Ε}^{2}=\int SE^{2}\left(\hat{\theta }|\theta \right)g\left(\theta \right)\mathrm{d\theta}. \end{array}$$

Unlike MLE, MAP and EAP estimation methods incorporate prior distributions for latent proficiency. In IRT, the latent‐proficiency distribution typically follows a standard normal distribution (or multivariate normal in M‐IRT) so that scores are expressed as standard deviations from the mean. Accounting for this prior information helps stabilize estimates, particularly for extreme and unexpected item‐score patterns.

The posterior probability evaluated at grid point $\theta_{q}$ for a person with response vector $x_{i}$ can be obtained by incorporating the prior distribution $g\left(\theta \right)$. Here, q=1,…,Q indices the quadrature points used to approximate the integral over the latent proficiency distribution. The posterior at grid point $\theta_{q}$ is given by

(15) $$\begin{array}{c} P\left(\theta_{q}|x_{i},\delta \right)=\frac{L\left(x_{i}|\theta_{q},\delta \right)g\left(\theta_{q}\right)}{\int L\left(x_{i}|\theta ,\delta \right)g\left(\theta \right)d\theta }. \end{array}$$

The probability $P\left(\theta_{q}|x_{i},\delta \right)$ is the probability of the union $x_{i}\cap \theta_{q}$ among all people with $x_{i}$.

MAP estimation consists of finding the latent proficiency level $\theta_{q}$ that maximizes the posterior probability $P\left(\theta_{q}|x_{i},\delta \right)$ for person i with response vector $x_{i}$. As with MLE, the standard error of the MAP estimator can be also approximated using Fisher information. However, since MAP incorporates prior information about the distribution of θ, the prior is also accounted for when estimating SE of MAP estimators:

(16) $$\begin{array}{c} \mathrm{SE}\left({\overset{˘}{\theta }}_{i}|\theta_{i}\right)=\sqrt{I_{\text{Posterior}}^{-1}\left(\theta_{i}\right)}, \end{array}$$

where $I_{\text{Posterior}}\left(\theta_{i}\right)$ denotes the posterior information about $\theta_{i}$, computed as $I\left(\theta_{i}\right)+{\left[\sigma_{\theta }^{2}\right]}^{-1}$, where $\sigma_{\theta }^{2}$ denotes the variance of the population distribution of θ, that is, $\sigma_{\theta }^{2}=1$. Similar to MLE estimators, this formulation generalized to M‐IRT, where $I_{\text{Posterior}}\left(\theta_{i}\right)=I\left(\theta_{i}\right)+\Sigma^{-1}$ and Σ denotes the populational trait covariance matrix. Notably, when prior information is uninformative (i.e. the prior distribution is uniform), MAP and MLE estimates are equivalent.

The EAP method estimates the proficiency level of a person with item‐score vector $x_{i}$ by computing the expected value of the posterior distribution of *θ* by approximation using a finite set of quadrature points $\theta_{q}$ as follows:

(17) $$\begin{array}{c} {\tilde{\theta }}_{i}=\sum_{q}\theta_{q}\cdot P\left(\theta_{q}|x_{i},\delta \right). \end{array}$$

Unlike MAP, EAP is less computationally demanding and can estimate scores that were not explicitly explored in the quadrature set. The standard error of EAP estimates (${\mathrm{SE}}_{\mathrm{EAP}}$), known as the posterior standard deviation of estimates, is approximated by the standard deviation of the expected estimate among individuals with the same response pattern:

(18) $$\begin{array}{c} \mathrm{SE}\left({\tilde{\theta }}_{i}|x_{i}\right)=\sqrt{\sum_{q}{\left(\theta_{q}-{\tilde{\theta }}_{i}\right)}^{2}\cdot P\left(\theta_{q}|x_{i},\delta \right)}. \end{array}$$

Unlike MLE and MAP, an empirical sample standard error of EAP is estimated as the square root of the average error variance across the observed item‐score vectors:

(19) $$\begin{array}{c} {\bar{\sigma }}_{Ε}^{2}=N^{-1}\sum_{i}SE^{2}\left({\tilde{\theta }}_{i}|x_{i}\right). \end{array}$$

Under standard regularity conditions, the sampling distribution of the MLE ${\hat{\theta }}_{i}$ is asymptotically normal around the true latent proficiency $\theta_{i}$ (Cramér, 1946, p. 500). In the Bayesian framework, when the number of items is sufficiently large, the posterior distribution of $\theta_{i}$ becomes sharply peaked around the Bayesian estimate (MAP or EAP).

The probability of an individual truly classified above a threshold corresponds to the cumulative area of the latent‐variable distribution at or above the classification threshold. Through Laplace's method, this posterior distribution, in the case of MAP and EAP estimators, can be approximated as normal, with mean equal to the estimated latent‐variable level and standard deviation given by the corresponding standard error of the estimator. Therefore, mastery probabilities in IRT‐based classification can be calculated by applying Equations (10) and (11), replacing ${\ddot{T}}_{i}$ and $T_{i}$ with latent proficiency estimator and true value, respectively, and defining thresholds on the latent variable scale. The extension to the multidimensional case defines the probability of multi‐attribute classifications from the integration region determined by whether each coordinate lies below or above its corresponding mastery threshold.

## 3 COGNITIVE DIAGNOSIS MODELS

CDMs focus on making data‐driven decisions about an individual's mastery or non‐mastery of specific attributes, based on criteria set in the Q‐matrix specifications. Mastery thresholds are estimated during the item calibration process, yielding categorical classifications rather than continuous score estimates. We assume that these classifications reflect the same underlying classification process as presented for CTT and IRT, where a true score or a latent variable is evaluated against a mastery threshold, but the continuous components are under‐identified in CDMs and therefore cannot be directly estimated. CDM score estimation yields probabilities of latent classes for each person, with each class defined by a combination of discrete attribute classifications. Unlike in CTT and IRT, where classification probabilities are computed given a proficiency estimator and its standard error, CDM‐based probabilities cannot be traced back to unique continuous scores. This implies that different combinations of continuous proficiency estimates and their standard errors may yield the same probability of latent class membership.

For simplicity, we focus on dichotomous attributes (e.g. de la Torre, 2011; Junker & Sijtsma, 2001) but CDMs for polytomous attributes have also been proposed (e.g. Chen & de la Torre, 2013; de la Torre et al., 2025). The three primary scoring methods, MLE, MAP and EAP, are modified to suit the discreteness of latent attributes in CDMs and provide three types of scores: (1) a classification at the latent class level representing an individual's profile or attribute combination, such as ${\overset{˘}{\alpha }}_{i}$; (2) a classification at the attribute level, such as ${\overset{˘}{\alpha }}_{i,k}$, indicating mastery or non‐mastery of each specific attribute indexed k, k=1,…,K (K is the total number of attributes); and (3) the expectancy of mastery of each specific attribute, expressed as the posterior probability of mastery, such as $P\left({\overset{˘}{\alpha }}_{i,k}=1|x_{i},\delta \right)$. In the CDM framework, ${\hat{\alpha }}_{i}$ and ${\overset{˘}{\alpha }}_{i}$ can be used to denote the MLE and MAP estimators of the attribute profile (i.e. the latent class with the highest likelihood or posterior probability). In contrast, ${\hat{\alpha }}_{i,k}$ and ${\overset{˘}{\alpha }}_{i,k}$ represent the modal values of the marginal expectation for attribute k across all classes under MLE and MAP estimation. To begin, we define the likelihood function for CDMs as

(20) $$\begin{array}{c} L\left(x_{i}|\alpha_{l},\delta \right)=\prod_{j=1}^{J}P\left(x_{i,j}|\alpha_{l},\delta \right), \end{array}$$

where $\alpha_{l}$ refers to the attribute profile vector representing latent class *l*, for *l* = 1, …, $2^{K}$, for dichotomous attributes. For example, $\alpha_{1}=\left(0,0,0\right)$ is the latent class that represents the non‐mastery of all three attributes in a given test.

The MLE of $\alpha_{i}$ given the item‐score vector $x_{i}$ involves identifying the $\alpha_{l}$ that maximizes the likelihood $L\left(x_{i}|\alpha_{l},\delta \right)$. The main differences between CDMs and IRT arise in quantifying measurement error and reliability. Because the item Fisher information cannot be computed for discrete attributes, theoretical measurement error and reliability indices cannot be predicted solely from item parameters but depend on specific item‐score vectors. Theoretical expectations of measurement error and reliability are approximated accounting for the full set of $2^{J}$ possible response vectors for the J items, while their empirical counterparts rely solely on the item‐score vectors observed in the dataset.

As with IRT, MAP and EAP estimators in CDMs incorporate prior information about the distribution of latent attributes. However, in this case, the prior distribution $P\left(\alpha_{l}\right)$, which represents the joint distribution of the attribute vectors, is not arbitrary. Rather, it is also estimated using empirical Bayes during the calibration of the item parameters as an additional step in the marginal maximum likelihood estimation process (de la Torre, 2009). The posterior probability of each latent class for individuals with item score pattern $x_{i}$ is defined as

(21) $$\begin{array}{c} P\left(\alpha_{l}|x_{i},\delta \right)=\frac{L\left(x_{i}|\alpha_{l},\delta \right)P\left(\alpha_{l}\right)}{\sum_{m}L\left(x_{i}|\alpha_{m},\delta \right)P\left(\alpha_{m}\right)}, \end{array}$$

where the numerator reflects the joint probability of having item‐score pattern $x_{i}$ and being in latent class $\alpha_{l}$, whereas the denominator represents the marginal probability of observing item‐score vector $x_{i}$ across all $2^{K}$ latent classes, as in,

(22) $$\begin{array}{c} P\left(x_{i}|\delta \right)=\sum_{m}L\left(x_{i}|\alpha_{m},\delta \right)P\left(\alpha_{m}\right). \end{array}$$

The MAP estimator ${\overset{˘}{\alpha }}_{i}$ given the item score vector $x_{i}$ is the latent class (sometimes, multiple latent classes when the Q‐matrix is unidentified) with the maximum posterior probability $P\left(\alpha_{l}|x_{i},\delta \right)$. Furthermore, $P\left(\alpha_{l}|x_{i},\delta \right)$ represents the probability of item‐score‐level diagnosis or classification of person *i* in class *l*. For example, in a three‐attribute test, ${\overset{˘}{\alpha }}_{i}=\left(1,0,0\right)$ indicates that person *i* has been diagnosed to have mastered the first attribute, but not the second and third attributes. From Equation (24), $P\left(\alpha_{l}|x_{i},\delta \right)$ can be interpreted in frequentist terms as the proportion of respondents with item score pattern $x_{i}$ whose true latent class is $\alpha_{l}$.

In addition to the item‐score‐level estimate, the attribute‐level estimate can also be computed. In particular, the marginal posterior probability of mastery at the attribute level, $P\left(\alpha_{k}=1|x_{i},\delta \right)$, can be computed by marginalizing $P\left(\alpha_{l}|x_{i},\delta \right)$ across all attribute patterns where $\alpha_{k}=1$; the marginal probability of non‐mastery $P\left(\alpha_{k}=0|x_{i},\delta \right)$ can be obtained in the same manner. Consistent with the interpretation of $P\left(\alpha_{l}|x_{i},\delta \right)$, the marginal probabilities $P\left(\alpha_{k}=1|x_{i},\delta \right)$ can be understood in frequentist terms as the proportion of respondents with item score pattern $x_{i}$ who truly master attribute *k*. The posterior probability $P\left(\alpha_{l}|x_{i},\delta \right)$ serves as an indicator of certainty of the classification of person *i* into a given latent class. Similarly, $P\left(\alpha_{k}|x_{i},\delta \right)$ serves as an indicator of certainty of the diagnosis of person *i* with respect to attribute *k*. These probabilities are EAP estimates and can then be discretized to obtain the EAP‐based mastery classification. Specifically, each probability is compared against a pre‐defined threshold. For instance, a threshold of .5 can be used to arrive at the classification ${\tilde{\alpha }}_{i,k}$ = 1, as in, person *i* is deemed to have mastered attribute *k* if $P\left(\alpha_{k}=1|x_{i},\delta \right)>.5$; ${\tilde{\alpha }}_{i,k}$ = 0, otherwise. In some applications, instead of a binary 0/1 classification with respect to an attribute, a region of uncertainty (e.g. $.4<P\left(\alpha_{k}=1|x_{i},\delta \right)<.6$) can be defined. The choice of thresholds reflects the amount of evidence required before a mastery or nonmastery classification can be made and may depend of how the resulting classifications will be used.

With an identified Q‐matrix, each item‐score vector has an associated unique latent class that maximizes the posterior probability. Consequently, each item‐score vector is associated with a unique MAP estimator, ${\overset{˘}{\alpha }}_{m}$. At the population level, a conditional classification matrix can be constructed by marginalizing the posterior probability $P\left(\alpha_{l}|x_{i},\delta \right)$ over all possible item score vectors whose MAP classification corresponds to ${\overset{˘}{\alpha }}_{m}$, where *m* indexes each possible latent class (Iaconangelo, 2017). At the sample level, an empirical approximation can be obtained from the observed item‐score vectors. Specifically, the estimated probability of belonging to true latent class $\alpha_{l}$ among those classified as ${\overset{˘}{\alpha }}_{m}$ is:

(23) $$\begin{array}{c} P\left(\alpha_{l}|{\overset{˘}{\alpha }}_{m},X\right)=\frac{\sum_{i}^{N}P\left(\alpha_{l}|x_{i},\delta \right)\cdot I\left({\overset{˘}{\alpha }}_{i}=\alpha_{m}\right)}{\sum_{i}^{N}P\left(\alpha_{l}|x_{i},\delta \right)}, \end{array}$$

where *N* denotes the sample size and $I\left(\cdot \right)$ is an indicator function denoting whether person *i* has been classified into latent class $\alpha_{m}$.

Similarly, a normalized contingency matrix can be constructed from estimated joint probabilities of true and estimated classifications:

(24) $$\begin{array}{c} P\left(\alpha_{l},{\overset{˘}{\alpha }}_{m}|X\right)=\frac{\sum_{i}^{N}P\left(\alpha_{l}|x_{i},\delta \right)\cdot I\left({\overset{˘}{\alpha }}_{i}=\alpha_{m}\right)}{N}, \end{array}$$

whose trace reflects the expected proportion of correct classification at the latent‐class level. This matrix can be further marginalized to reflect estimated joint probabilities of attribute‐level mastery by summing across latent classes with or without mastery of attribute *k*, or computed directly as (Wang et al., 2015):

(25) $$\begin{array}{c} P\left(\alpha_{k}=0,{\overset{˘}{\alpha }}_{k}=0|X\right)=N^{-1}\sum_{i=1}^{N}I\left({\overset{˘}{\alpha }}_{\mathrm{ik}}=0\right)P\left(\alpha_{k}=0|x_{i},\delta \right), \end{array}$$

$$P\left(\alpha_{k}=0,{\overset{˘}{\alpha }}_{k}=1|X\right)=N^{-1}\sum_{i=1}^{N}I\left({\overset{˘}{\alpha }}_{\mathrm{ik}}=1\right)P\left(\alpha_{k}=0|x_{i},\delta \right),$$

$$P\left(\alpha_{k}=1,{\overset{˘}{\alpha }}_{k}=0|X\right)=N^{-1}\sum_{i=1}^{N}I\left({\overset{˘}{\alpha }}_{\mathrm{ik}}=0\right)P\left(\alpha_{k}=1|x_{i},\delta \right),\text{and}$$

$$P\left(\alpha_{k}=1,{\overset{˘}{\alpha }}_{k}=1|X\right)=N^{-1}\sum_{i=1}^{N}I\left({\overset{˘}{\alpha }}_{\mathrm{ik}}=1\right)P\left(\alpha_{k}=1|x_{i},\delta \right).$$

Table 1 provides an example of the estimated joint cross‐classification table at the attribute level. In here, the calculation of row marginals would reflect the estimated probability of mastery/non‐mastery of attribute *k*, whereas column marginals would denote the proportion of estimated mastery status in the sample.

**TABLE 1:** Example of populational joint cross‐classification matrix at the attribute level.

		${\overset{˘}{\alpha }}_{k}$	
		{0}	{1}
$a_{k}$	{0}	.63	.05
	{1}	.02	.30

Compared to classification in CTT, assuming a continuous variable underlying each discrete attribute, the joint probability of $\alpha_{k}$ and ${\overset{˘}{\alpha }}_{k}$ for the *k*th attribute corresponds to the integration over a rectangular region of the bivariate normal distribution defined by the true scores and their respective estimates relative to the mastery threshold. For instance, $P\left(\alpha_{k}=1,{\overset{˘}{\alpha }}_{k}=1\right)=P\left(T_{k}>\tau_{k},{\ddot{T}}_{k}>\tau_{k}\right)$ represents the probability in the region where both the true score $T_{k}$ and its estimator ${\ddot{T}}_{k}$ exceed the threshold $\tau_{k}$. In IRT, applying Laplace's approximation for Bayesian estimates, the analogous probability $P\left(\alpha_{k}=1,{\overset{˘}{\alpha }}_{k}=1|X\right)=P\left(\theta_{k}>\tau_{k},{\overset{˘}{\theta }}_{k}>\tau_{k}|X\right)$ corresponds to the integration over the rectangular region of the bivariate normal distribution of $\theta_{k}$ and ${\overset{˘}{\theta }}_{k}$ above the threshold along each coordinate.

## 4 RELIABILITY OF CLASSIFICATIONS

A wide variety of indices to quantify the correctness of classifications can be derived from the conditional and joint classification matrices. These indices are often categorized as assessing classification accuracy, which reflects the agreement between observed and true classifications; classification consistency, which is the agreement between classifications derived from different tests; and reliability, which is often defined from the linear relationship between true and observed scores. For a comprehensive overview, see Sinharay and Johnson (2019).

Templin and Bradshaw (2013) proposed estimating the reliability of posterior probabilities of mastery using the squared tetrachoric correlation ($\rho_{\mathrm{tet}}$) derived from the attribute cross‐classification matrix. Conceptually, this coefficient represents the reliability of hypothetical continuous variables underlying both observed and true classifications for a given attribute *k*. Expanding on this idea, Johnson and Sinharay (2020) introduced an alternative approach by defining the point biserial correlation ($\rho_{\mathrm{bis}}$) between true mastery status and the posterior probability of mastery as:

(26) $$\begin{array}{c} \rho_{\mathrm{bis}}=\frac{P\left(\alpha_{k}=1,{\overset{˘}{\alpha }}_{k}=1|X\right)}{P\left(\alpha_{k}=1|X\right)}-\frac{P\left(\alpha_{k}=0,{\overset{˘}{\alpha }}_{k}=1|X\right)}{P\left(\alpha_{k}=0|X\right)}. \end{array}$$

Notably, the squared $\rho_{\mathrm{tet}}$ is expected to be equal to or greater than $\rho_{\mathrm{bis}}$, as $\rho_{\mathrm{tet}}$ captures the association between two continuous variables, whereas $\rho_{\mathrm{bis}}$ reflects the relationship between a continuous and a discrete variable. In turn, $\rho_{\mathrm{bis}}$ will be equal to or greater than the true reliability of classifications, which quantifies the association between the two discrete variables (i.e., $\alpha_{k}$ and ${\overset{˘}{\alpha }}_{k}$). Given these hierarchical relationships, caution is warranted when interpreting squared $\rho_{\mathrm{bis}}$ and $\rho_{\mathrm{tet}}$ in contexts where the primary focus is on categorical classification rather than probability‐based estimates.

As noted in the previous sections, posterior probabilities of mastery for each attribute do not provide a direct ordering of underlying proficiencies. Instead, they depend jointly on both the proficiency level and the standard error of its estimator. In practice, two examinees with identical true proficiency may exhibit different mastery probabilities depending on the estimation error. Consequently, comparisons among examinees should rely on categorical classifications rather than posterior probabilities, since ordering by probabilities does not guarantee an ordering by proficiency.

Accordingly, reliability coefficients in CDMs should quantify the precision of classification outcomes and not the precision of their posterior probabilities (Hambleton, 1983; Schauber & Hecht, 2020). Moreover, if decisions concern profiles referring to combination rather than mastery of single attributes, reliability should be evaluated at the class level, whereas attribute‐level interpretations call for attribute‐specific reliability.

In the next section, we propose a more unified psychometric perspective in which reliability coefficients for classifications across CTT, IRT and CDMs are anchored in the coefficient of determination and can be framed within the general reliability framework outlined by Liu et al. (2025). This unified approach facilitates extending methodologies traditionally applied in CTT and IRT to CDMs, enabling practical applications such as correcting validity coefficients for attenuation due to unreliability.

Although defining reliability as the squared correlation between true and observable scores is not a new idea (see Lord & Novick, 1968), previous works in CDMs have primarily focused on the reliability of posterior mastery probabilities (e.g. based on tetrachoric or point‐biserial correlations, see Sinharay & Johnson, 2019) rather than the reliability of the resulting discrete classifications. The choice of reliability indices should, however, align with the intended use of the test scores (AERA, APA and NCME, 2014). In the context of cognitive diagnostic assessments, decisions are often based on mastery classifications rather than on the posterior mastery probabilities per se. Therefore, indices that quantify the reliability of the classifications provide evidence regarding the precision of these decision rules and, thus, contribute to the justification of their intended use (Kane, 2013).

For binary mastery classifications at the attribute level, the population coefficient of determination $R^{2}$, as formulated next in Equation (27), simplifies to the squared value of Yule's (1912) ϕ‐coefficient. The population reliability parameter is defined as

(27) $$\begin{array}{c} R^{2}=\frac{\mathrm{Cov}{\left(\alpha_{k},{\overset{˘}{\alpha }}_{k}\right)}^{2}}{\mathrm{Var}\left(\alpha_{k}\right)\mathrm{Var}\left({\overset{˘}{\alpha }}_{k}\right)}, \end{array}$$

where all expectations, variances and covariances concern the population joint distribution of the true attribute mastery indicator $\alpha_{k}$ and its estimated counterpart ${\overset{˘}{\alpha }}_{k}$. In particular,

$$\mathrm{Cov}\left(\alpha_{k},{\overset{˘}{\alpha }}_{k}\right)=E\left(\alpha_{k,}{\overset{˘}{\alpha }}_{k}\right)-E\left(\alpha_{k}\right)E\left({\overset{˘}{\alpha }}_{k}\right)=P\left(\alpha_{k}=1,{\overset{˘}{\alpha }}_{k}=1\right)-P\left(\alpha_{k}=1\right)P\left({\overset{˘}{\alpha }}_{k}=1\right),$$

$$\mathrm{Var}\left(\alpha_{k}\right)=P\left(\alpha_{k}=1\right)\cdot P\left(\alpha_{k}=0\right),\text{and}\;\mathrm{Var}\left({\overset{˘}{\alpha }}_{k}\right)=P\left({\overset{˘}{\alpha }}_{k}=1\right)\cdot P\left({\overset{˘}{\alpha }}_{k}=0\right).$$

Next, the population coefficient of determination can be expressed equivalently as follows:

(28) $$\begin{array}{c} R^{2}=\frac{{\left(\mathrm{ad}-\mathrm{bc}\right)}^{2}}{\left(a+b\right)\left(c+d\right)\left(a+c\right)\left(b+d\right)}=\phi^{2}. \end{array}$$

where *a*, *b*, *c* and *d* denote the population joint probabilities defining the cross‐classification of true and estimated mastery states:

(29) $$\begin{array}{c} a=P\left(\alpha_{k}=0,{\overset{˘}{\alpha }}_{k}=0\right),\;b=P\left(\alpha_{k}=0,{\overset{˘}{\alpha }}_{k}=1\right) \end{array}$$

$$c=P\left(\alpha_{k}=1,{\overset{˘}{\alpha }}_{k}=0\right),\text{and}\;d=P\left(\alpha_{k}=1,{\overset{˘}{\alpha }}_{k}=1\right).$$

In practice, these population probabilities are unknown and are replaced by sample‐based estimators obtained from the entries in the estimated joint cross‐classification matrix at the attribute level. Because $\phi^{2}$ offers the reliability of classification, it is generally expected to be lower than the squared biserial correlation and squared tetrachoric correlation coefficients computed from the joint cross‐classification matrix.

To extend this coefficient from the attribute to the class level, we estimate the joint cross‐classification probabilities for attribute vector α in class *l* using the estimated binary indicators of class assignment, $I\left({\overset{˘}{\alpha }}_{i}=l\right)$ or $I\left({\overset{˘}{\alpha }}_{i}\neq l\right)$, and estimated posterior probability of membership or non‐membership to class *l*; that is, $P\left(\alpha =l|x_{i},\delta \right)$ and $P\left(\alpha \neq l|x_{i},\delta \right)$:

(30) $$\begin{array}{c} P\left(\alpha \neq l,\overset{˘}{\alpha }\neq l\right|X)=N^{-1}\sum_{i=1}^{N}I\left({\overset{˘}{\alpha }}_{i}\neq l\right)P\left(\alpha \neq l|x_{i},\delta \right), \end{array}$$

$$P\left(\alpha \neq l,\overset{˘}{\alpha }=l|X\right)=N^{-1}\sum_{i=1}^{N}I\left({\overset{˘}{\alpha }}_{i}=l\right)P\left(\alpha \neq l|x_{i},\delta \right),$$

$$P\left(\alpha =l,\overset{˘}{\alpha }\neq l|X\right)=N^{-1}\sum_{i=1}^{N}I\left({\overset{˘}{\alpha }}_{i}\neq l\right)P\left(\alpha =l|x_{i},\delta \right),\;\text{and}$$

$$P\left(\alpha =l,\overset{˘}{\alpha }=l|X\right)=N^{-1}\sum_{i=1}^{N}I\left({\overset{˘}{\alpha }}_{i}=l\right)P\left(\alpha =l|x_{i},\delta \right).$$

From this, an $R^{2}$ value can be computed as an estimator of the squared correlation between true and estimated class assignments.

Delta‐method approximations may be used to obtain analytical standard errors of $R^{2}$ in large sampless, treating the estimated joint probabilities as asymptotically normal. For similar developments for reliability in IRT, see Andersson and Xin (2018), Sung and Liu (2026) and for reliability, see van der Ark (2025). In small samples, resampling‐based procedures such as nonparametric bootstrap methods provide a flexible and implementation‐friendly approach for constructing confidence intervals for $R^{2}$.

When assessment decisions are made at the attribute level (e.g. providing remedial materials for specific skills), the appropriate index is $\phi^{2}\left(\alpha_{k},{\overset{˘}{\alpha }}_{k}\right)$, which reflects the proportion of variance in estimated classifications attributable to true attribute mastery. Conversely, when decisions are based on latent classes defined by mastery profiles across multiple attributes, estimated $\phi^{2}\left(\alpha_{l},{\overset{˘}{\alpha }}_{l}\right)$ should be reported, as it reflects the proportion of variance in estimated class membership attributable to true class membership. To facilitate the implementation of the formulations presented in the previous sections, accompanying open‐source code is provided at https://osf.io/ygh7p.

## 5 DISCUSSION

We expressed mastery probabilities and class probabilities, framed differently in CTT, IRT and CDMs, in a common probabilistic framework. Despite differences in the underlying models, each framework provides a probability statement about whether a true latent value exceeds a threshold or belongs to a specific class. The unified reliability indices capture the association between true and estimated classifications. Framing reliability as the squared correlation between true and estimated mastery or class membership provides a measure that is interpretable across frameworks and adaptable to the purpose of the assessment. Whether decisions are made at the attribute level (e.g. identifying skill mastery), the profile level (e.g. diagnosing combinations of skills) or the score level (e.g. ranking individuals on a continuum), reliability can be reported using a consistent metric that reflects the precision of classification.

Our approach clarifies the conceptual link between frameworks traditionally treated as distinct, showing that CDM classification probabilities are formally related to the sampling or posterior distributions central to CTT and IRT. In addition, it promotes comparability in practice: reliability estimates no longer depend exclusively on the framework used, but instead on how well observed data reproduce true classifications or mastery states. Several issues require further study. The indices developed here are analytically valid at the population level, but their finite‐sample behaviour remains unknown and needs separate attention.

## DISCLOSURE OF ARTIFICIAL INTELLIGENCE–GENERATED CONTENT (AIGC) TOOLS

No generative AI tools were used in the creation of this article.

## AUTHOR CONTRIBUTIONS

**Jimmy de la Torre:** Conceptualization; methodology; writing – review and editing; supervision; investigation. **Klaas Sijtsma:** Conceptualization; investigation; methodology; writing – review and editing; supervision. **Rodrigo Schames Kreitchmann:** Conceptualization; methodology; investigation; software; writing – review and editing; writing – original draft.

## CONFLICTS OF INTEREST STATEMENT

The authors have no conflicts of interest to declare.

## Data Availability

This study presents theoretical and methodological advancements. Open‐source code to implement the formulations discussed is available on the Open Science Framework (OSF) at https://osf.io/ygh7p.
